# A System of Obstetrics

**Published:** 1888-08

**Authors:** 


**A System of Obstetrics.** By American authors. Edited by BARTON COOKE HIRST, M. D., Associate Professor of Obstetrics in the University of Pennsylvania, etc., etc. Volume I. Illustrated with a colored plate and three hundred and nine engravings on wood. Philadelphia: Lea Brothers & Co. 1888.

The contents of this volume are: 1st—History of Obstetrics, by George J. Engelmann, M. D. 2d—The Physiology and

Histology of Ovulation, and Menstruation, and Fertilization, the Development of the Embryo. By H. Newell Martin, F. R. S., M. D. 3d—The Fœtus, its Development, Anomalies, Monstrosities, Diseases and Premature Expulsion. By Barton Cooke Hirst, M. D. 4th—Pregnancy: its Physiology, Pathology, Signs and Differential Diagnosis. By William Wright Jaggard, M. D. 5th—The Conduct of Labor and the Management of the Puerperal State. By Samuel C. Busey, M. D. 6th—On the Mechanism of Labor and the Treatment of Labor based on the Mechanism. By R. A. F. Penrose, M. D. 7th—The Use of Anæsthetics in Labor. By J. C. Reeve, M. D. 8th—Anomalies of the Forces in Labor. By Theophilus Parvin, M. D. This work is intended to occupy a similar position in the science and art of obstetrics, as its companion system of gynecology by American authors. The array of eminent writers, the names of whom and their subjects we note above, give rise to large expectations as to the high character of the work. In this volume we have much to commend, and we think the venture in its publication was fully justified. The treatment of the subject is different from the ordinary treatise on obstetrics, and this peculiarity adds value to the work The views of the contributors conflict in some instances. This gives a strong character and increased value to the work. We commend it to our readers.

